# Hepatocellular Carcinoma in Africa: Epidemiology, Prevention and Outcomes

**DOI:** 10.1007/s11901-026-00744-8

**Published:** 2026-07-15

**Authors:** Joseph A Akambase, Ponsiano Ocama, Jose D. Debes

**Affiliations:** 1 Department of Medicine, Hennepin Healthcare, Minneapolis, MN USA; 2 Department of Medicine, Makerere University College of Health Sciences, Kampala, Uganda; 3 Infectious Disease Institute, Makerere University, Kampala, Uganda; 4 Department of Medicine, University of Minnesota, Minneapolis, MN 55455 USA; 5 Department of Gastroenterology, Erasmus MC, Rotterdam, The Netherlands

**Keywords:** Hepatocellular carcinoma, Global health, Africa, Liver cancer, North Africa, Sub-Saharan Africa, Hepatitis B virus, Hepatitis C virus, Aflatoxin, Metabolic dysfunction–associated steatotic liver disease, Cancer prevention, Cancer surveillance

## Abstract

**Purpose of review:**

This review synthesizes the current knowledge of hepatocellular carcinoma (HCC) epidemiology across Northern and sub-Saharan Africa. We examine how causes of HCC differ by region, highlight prevention and early detection strategies, and identify gaps in treatment and palliative care to improve outcomes.

**Recent findings:**

The causes of HCC vary markedly across the continent. HCV predominates in Northern Africa, whereas HBV infection and dietary aflatoxin exposure prevail in sub-Saharan Africa. Rising obesity, diabetes, harmful alcohol use, and hepatitis D co-infection are important but under-researched contributors. Most patients still present with advanced disease, fewer than 6% receive curative therapy, median survival is measured in months, and access to palliative care remains dismal.

**Summary:**

HCC poses a major challenge in Africa, marked by high incidence, early age at diagnosis, and among the worst survival outcomes worldwide. Primary prevention through HBV birth-dose vaccination, aflatoxin mitigation, and population-level HCV treatment offers the most effective way to reduce mortality, yet implementation barriers persist.

## Introduction

Primary liver cancer is the sixth most commonly diagnosed malignancy and the third leading cause of cancer‑related death globally, with approximately 900,000 new cases and over 750,000 deaths reported in 2022 [1]. Hepatocellular carcinoma (HCC) constitutes over 85% of primary liver cancers and carries a nearly one-to-one mortality‑to‑incidence ratio worldwide [1, 2]. Although HCC is a global concern, its impact is predominantly observed in resource-limited regions, particularly in East Asia and Africa [3]. The overwhelming majority of HCC occurs in the setting of chronic liver disease. This has major implications in the distinct epidemiology of HCC worldwide, as the frequency of specific liver disease of different regions dictates the frequency and biology of the cancer [3]. The etiologic landscape of HCC in Africa is notably diverse [4]. In Northen Africa, Hepatitis C Virus (HCV) infection dominates, a pattern that reflects a passed era of likely transmission during mass anti-schistosomiasis campaigns in Egypt and neighboring states [5, 6]. Across SSA, chronic Hepatitis B (HBV) infection is the major cause of HCC. Indeed, HBV infection combined with lifelong dietary exposure to aflatoxin B1 resulting in HCC at a median age one to two decades younger than in other settings [4, 7, 8]. Co-infection with other viruses alters the epidemiology of HCC in the continent. Hepatitis D virus (HDV), a parasite virus that needs HBV to replicate, exacerbates the progression of fibrosis, and it is estimated to be responsible for approximately 10–20% of HBV-related HCC cases in the region [9, 10]. Concurrent infection with the human immunodeficiency virus (HIV) further increases the risk of HCC [11, 12]. 

In addition to infection and environmental toxins, the increasing trends of urbanization, demographic transition and the adoption of Western dietary habits over the last decades have led to the emergence of metabolic dysfunction-associated steatotic liver disease (MASLD) as well as hazardous alcohol consumption as significant contributing factors for HCC in the continent [7, 13–15]. 

This review synthesizes current evidence on the epidemiology of HCC in Northern and sub-Saharan Africa, the state of primary and secondary prevention, and the outcomes, technological innovations, persistent gaps and palliative-care needs that will define progress over the next decade.

### Epidemiology of HCC in Northern Africa and Egypt

In Northern Africa, particularly in Egypt, HCC continues to manifest some of the highest incidence rates globally. Studies report age‑standardized incidence rates of 13–15 per 100,000, with HCC remaining the predominant cause of cancer death [1, 16, 17]. The epidemiology of HCC in this region cannot be separated from the history of parenteral anti-schistosomiasis therapy. From the 1920s to the early 1980s, millions of injections were thought to be administered using inadequately sterilized needles, resulting in the largest global recorded iatrogenic HCV epidemic [5, 6]. By 2015 the Egyptian Demographic and Health Survey estimated a viremic prevalence of approximately 6–10% among adults, the highest globally [17, 18]. 

In the Africa Liver Cancer Consortium multicenter study involving 2,566 patients, HCV was identified as the cause of HCC in 84% of Egyptian patients with the median age at diagnosis being 58 years, approximately a decade older than patients in other regions of the continent [4]. In the Maghreb region comprising Tunisia, Morocco and Algeria, HCC arises from a combination of HBV, HCV and metabolic risk factors, with up to 60% of patients testing positive for anti‑HCV, 18% for HBsAg and nearly 20% of patients diagnosed with diabetes mellitus, a major contributor to MASLD [19]. Indeed, in the North African region, HBV, HCV and MASLD each contribute to approximately one third of HCC cases [15, 20]. 

**Fig. 1:**
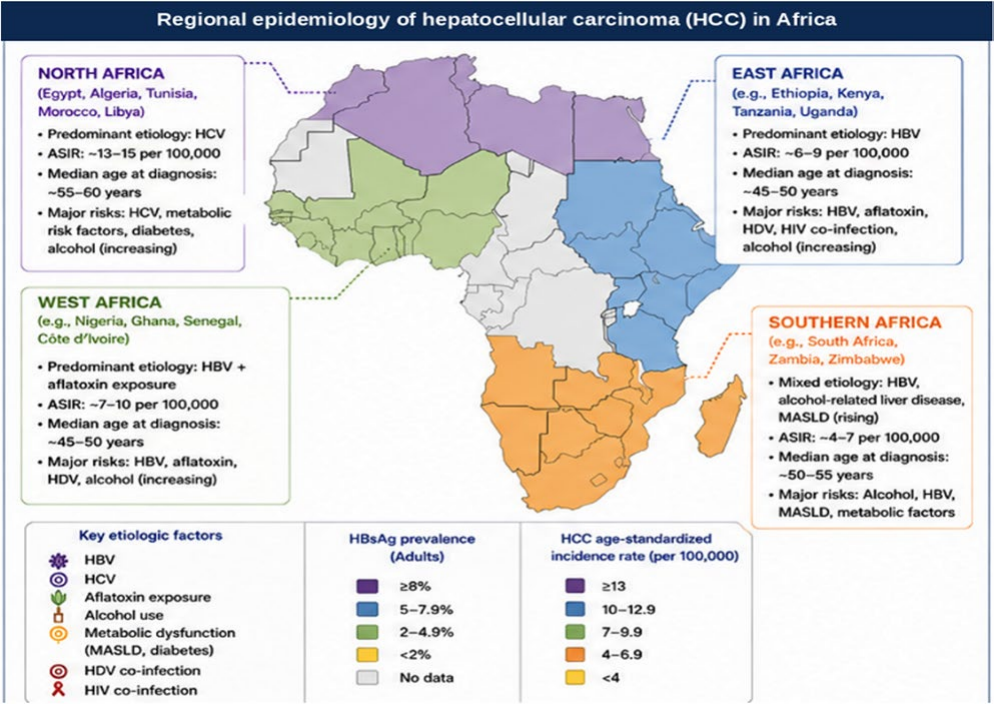
Regional epidemiologic heterogeneity of hepatocellular carcinoma across Africa. Four regional HCC patterns emerge. North Africa is HCV-dominant, with the highest incidence (ASIR ~13–15/100,000) and oldest age at diagnosis (55–60 years). West/East Africa remain HBV/aflatoxin-driven, presenting earlier (45–50 years). Southern Africa shows a mixed profile with rising alcohol-related disease and MASLD alongside endemic HBV. Shading depicts HBsAg seroprevalence and ASIR by region; icons denote dominant etiologies. Data from GLOBOCAN 2022, the Africa Liver Cancer Consortium, and regional studies; gray areas reflect missing registry data. ASIR, age-standardized incidence rate; HBV/HCV/HDV, hepatitis B/C/D virus; MASLD, metabolic dysfunction–associated steatotic liver disease

Egypt’s proactive and progressive approach to addressing the HCV epidemic has significantly altered the trajectory of liver disease on the continent. In 2014, the nation initiated a national elimination program, which has since evolved into the world’s largest population‑based screen‑and‑treat initiative globally. This program screened over 48 million adults, reducing active HCV prevalence by over 90% and positioning Egypt on track to meet the World Health Organization’s 2030 elimination targets [18, 21]. However, many Egyptians who have been cured of HCV already have advanced fibrosis or cirrhosis, leaving them at residual risk even post-cure [2, 13]. Simultaneously, rapid urbanization has led to epidemics of obesity, type 2 diabetes and MASLD, which are projected to become primary contributors to liver cancer in coming decades [14, 15, 22]. The Egyptian experience provides both optimism and caution, while the elimination of hepatitis is feasible, it is imperative to enhance surveillance and management of metabolic liver disease to ensure that efforts in virologic control translate into lower HCC mortality. The regional differences in HCC epidemiology and major etiologic drivers across Africa are summarized in Fig. 1.

### Epidemiology of HCC in Sub-Saharan Africa

Sub-Saharan Africa exhibits the highest prevalence of diverse and underreported HCC epidemiology in the continent. The age-standardized incidence rates, ranging from 4 to 11 per 100,000, likely underestimate the true burden by about 20–40% due to incomplete cancer registries and underdiagnosis [16, 23]. In several West African nations, including The Gambia, Guinea and Mali, HCC represents a primary cause of premature mortality [17]. A notable characteristic in SSA is early onset of disease: the median age at diagnosis ranges from 30 to 50 years, with nearly 40% of HBV‑related HCC cases occurring before the age 40, which is an almost decade earlier than observed in Asia or Europe [4, 8, 24]. 

Chronic HBV infection remains the primary etiologic factor, accounting for 55–70% of cases across SSA [4, 7, 17]. The prevalence of HBsAg in this region is estimated at 6.1% with large variations according to country and even regions within states [23, 25]. West and Central Africa remain hyperendemic, with HBsAg prevalence exceeding 8% in countries such as Nigeria, Burkina Faso, Gabon and Cameroon [2, 26]. East and Southern Africa display intermediate endemicity (2–8%), with reported prevalence around 3–6% in Kenya, Zambia, Côte d’Ivoire, Senegal and South Africa [2]. This marks stark differences with North African countries that generally sit at an HBV rate under 2% [2]. These differences closely align with the regional age-standardized incidence rates of HCC, which fall from 10 per 100,000 in West Africa to 7.5 per 100,000 in Central and East Africa and 6.5 per 100,000 in Southern Africa [23, 27]. (Fig. 1) The majority of infections are acquired perinatally or during early childhood. Exposure to aflatoxin B1 from improperly stored maize and groundnuts acts synergistically with HBV, inducing the well described TP53 R249S mutations and frequently leading to early HCC in non‑cirrhotic livers [4, 23, 28]. It is worth noting that most of the data on HCC from SSA comes from urban settings. A recent satellite spatial scaling analysis across 36 African towns and cities demonstrated that proximity to rural areas was associated with a 71% higher frequency of HBV-related HCC (relative risk 1.71, 95% CI 1.52–1.93), suggesting an under investigated incidence of HCC in rural areas of the continent [29]. Contributing factors to this disparity likely include lower HBV vaccination coverage, higher rates of home births, increased aflatoxin exposure from subsistence farming and inadequate grain storage, and the concentration of diagnostic and treatment infrastructure within major urban centers [29]. 

HBV-predominant HCC cohorts have been consistently reported in Nigeria, Ghana, Ethiopia, Senegal, The Gambia and South Africa [24, 30–35]. However, recent evidence from Nigeria suggests that HCV may represent a larger contributor to HCC risk than previously appreciated, particularly in the context of parental and healthcare-related transmission [11]. Co‑infections are important modifiers of HCC risk in SSA. HIV affects more than 25 million people in the region and is associated with an approximately fourfold higher risk of HCC, specifically in the setting of HBV or HCV co-infection [11, 12, 36]. HDV, although substantially underdiagnosed, represents an additional contributor to HBV-related HCC through its association with more aggressive liver disease and accelerated progression to cirrhosis and liver cancer [9, 10]. 

Two etiological factors warrant particular emphasis because they are rapidly increasing and remain consistently underreported across SSA: alcohol consumption and MASLD. Harmful alcohol consumption continues to rise, with median per capita pure alcohol intake estimated at 6.3 L annually in the WHO African region and substantially higher national levels reported in South Africa, Uganda, Namibia and Nigeria [23]. These estimates likely underestimate true alcohol exposure because they do not account for informal home-brewed and locally produced beverages, which remain common in rural communities and are rarely captured in national surveys [17, 23]. Traditional home-brewed beers are often fermented in iron containers, leading to high concentrations of bioavailable iron. This condition contributes to dietary iron overload, which is an independent risk factor for HCC and may further enhance the carcinogenic effects of HBV, aflatoxin exposure and MASLD [2, 23]. Locally brewed beverages have also been shown to contain aflatoxin B1 concentrations exceeding WHO safety thresholds [17]. 

At the same time, rapid urbanization and the ongoing nutrition transition are contributing to rising rates of overweight, obesity and type 2 diabetes across an expanding middle class, driven in part by sedentary lifestyles, increased sugar-sweetened beverage consumption and adoption of Western dietary patterns [17, 23]. The growing burden of MASLD in Africa is increasingly progressing to cirrhosis and HCC at rates comparable to those observed in Caucasian populations, challenging earlier assumptions of relative protection among Black African populations [17]. Few SSA countries have implemented structured public health or surveillance programs targeting harmful alcohol use or MASLD, and both risk factors remain poorly captured in regional cancer registries. Without coordinated intervention, these emerging epidemics may undermine the anticipated gains from HBV vaccination and HCV elimination efforts over the coming decades.

### Prevention and Early Detection

Primary prevention remains the most effective strategy for reducing HCC mortality in Africa. The implementation of universal HBV vaccination, together with perinatal prophylaxis, can significantly decrease HBV transmission and long-term risk of HCC [37]. Epidemiological studies indicate that achieving high coverage of hepatitis B vaccination could potentially reduce the incidence of HCC cases in the continent by up to 50% over the next three to five decades [38]. The potential impact of this approach is exemplified by Taiwan’s experience, where neonatal HBV vaccination reduced chronic HBV carriage from 10% to 0.5% and was associated with declines in HCC incidence and mortality of 80% and 63%, respectively [38, 39]. Unfortunately, implementation of vaccination across Africa remains inconsistent. Although regional coverage for the three-dose HBV vaccine series reached 76% by 2015, compared with a global average of 84%, only 10 of 47 African countries had incorporated the WHO-recommended birth-dose vaccine into their Expanded Program on Immunization [28, 40]. High rates of home deliveries, unreliable vaccine supply chains, and financial barriers continue to limit timely administration of the birth-dose vaccine. Consequently, only 1% of infants in Nigeria and The Gambia receive HBV vaccination within 24 h of birth, in contrast to 50% in Botswana [40]. These vaccination gaps disproportionately affect rural populations. In a rural Tanzanian screening cohort, 76% of participants were unaware of the need for HBV vaccination and 92% reported no prior vaccination history [41]. Any meaningful HBV and HCC elimination strategy in SSA must therefore expand prevention, screening and surveillance efforts beyond the academic referral centers where services are currently concentrated.

**Fig. 2:**
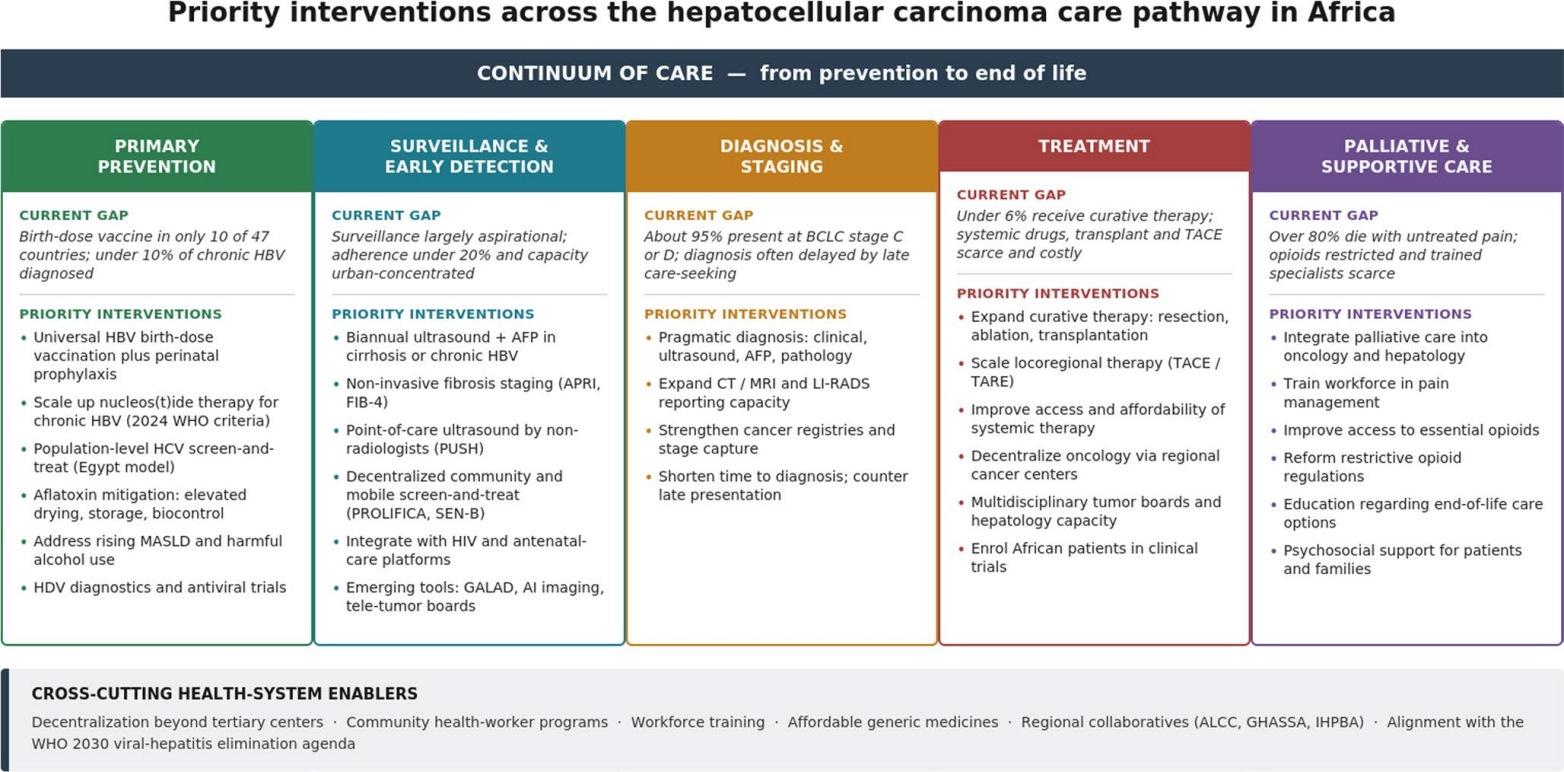
Priority interventions and current gaps across the HCC care continuum in Africa. For each care stage—prevention, surveillance, diagnosis/staging, treatment, palliative care—the dominant current gap is paired with priority interventions. Gaps include limited birth-dose HBV vaccination (10/47 countries), late-stage presentation (~95% BCLC C/D), and low curative-therapy uptake (<6%). Interventions range from established strategies (biannual ultrasound ± AFP, non-invasive fibrosis staging) to context-adapted tools (non-radiologist point-of-care ultrasound, GALAD score, tele-tumor boards) and system-level enablers (decentralization, workforce training, affordable generics). AFP, α-fetoprotein; APRI, AST-to-platelet ratio; BCLC, Barcelona Clinic Liver Cancer; FIB-4, fibrosis-4 index; LI-RADS, Liver Imaging Reporting and Data System; TACE/TARE, transarterial chemo-/radioembolization; ALCC, Africa Liver Cancer Consortium; GHASSA, Gastroenterology and Hepatology Association of Sub-Saharan Africa; IHPBA, International Hepato-Pancreato-Biliary Association

Beyond vaccination, expanding access to nucleoside analogue therapy for chronic HBV, is a cost-effective strategy that can significantly reduce long-term HCC risk. This is particularly pertinent in light of the expanded treatment thresholds recommended in the updated 2024 WHO guidelines [42]. Despite these advances, antiviral therapy remains inaccessible to many eligible individuals, and fewer than 10% of persons living with chronic HBV across the continent are aware of their diagnosis [26, 42]. Egypt’s HCV elimination program demonstrates that population-level viral control can be achieved in low- and middle-income settings through strong political commitment, affordable generic medications, and integrated screening and treatment infrastructure [18, 21]. Scaling similar approaches for HBV and HCV across SSA is now a strategic priority. Priority interventions across the HCC care pathway are summarized in Fig. 2. Meanwhile, HDV remains neglected; improved diagnostic capacity and new antiviral trials are needed [9]. 

Mitigating aflatoxin exposure constitutes an important component of preventive strategies. Effective interventions include drying maize and groundnuts on elevated platforms, improving household storage and utilizing non-toxigenic strains of Aspergillus to competitively displace toxin‑producing strains [23, 43]. When implemented alongside HBV vaccination, these aflatoxin control measures have the potential to significantly reduce HCC risk [4, 28]. Public health initiatives should also address MASLD and alcohol-related liver disease. Promoting healthy diets, regular physical activity and moderation in alcohol consumption could help reduce the future burden of non‑viral HCC.

In SSA, secondary prevention through the surveillance of high‑risk populations remains largely aspirational. International guidelines recommend semi‑annual abdominal ultrasound combined with serum α‑fetoprotein testing for patients with cirrhosis or chronic HBV infection, yet screening program are few and sparse. Ultrasound equipment and trained personnel are predominantly concentrated in tertiary urban centers, and adherence to the recommended semi-annual surveillance seldom exceeds 20% [44–46]. Key barriers include limited imaging capacity, workforce shortages, financial constraints, and low awareness among both patients and healthcare providers. Innovative decentralized models are beginning to emerge. Community- and mobile-based HBV and HCC screening programs in Tanzania have demonstrated the feasibility of integrating point-of-care HBsAg testing with portable ultrasound services [41, 47]. The Prevention of Liver Fibrosis and Cancer in Africa (PROLIFICA) program in The Gambia and Senegal previously established proof of concept for this approach, demonstrating that community-based HBV screen-and-treat is feasible, acceptable and cost-effective in West African settings [48]. The SEN-B prospective cohort in Senegal has further shown that HCC surveillance among HBsAg-positive individuals is achievable at scale in West Africa, with 93% adherence at 12 months and an HCC detection rate of 1.24 per 1,000 person-years [49]. The 2024 WHO simplified chronic HBV guidelines and encouraged simple non-invasive fibrosis scores, including the aspartate aminotransferase-to-platelet ratio index (APRI) and Fibrosis-4 (FIB-4) index in settings where transient elastography is unavailable [42]. Both scores can be calculated from routine laboratory tests at minimal cost [23, 42]. Point-of-care ultrasound performed by non-radiologists, as demonstrated in the PUSH protocol, represents a pragmatic complementary strategy that can identify cirrhosis and focal liver lesions at the primary care level, while guiding referral for HCC surveillance in settings where conventional imaging is unavailable [50]. Addressing these prevention gaps will require integrated strategies embedded within primary care, aligned with the WHO’s 2030 viral-hepatitis elimination agenda.

Several collaborative initiatives are currently engaged in addressing deficiencies related to HCC surveillance. The Africa Liver Cancer Consortium, the Gastroenterology and Hepatology Association of sub-Saharan Africa (GHASSA), and the 2023 Africa HCC management guidelines offer an important platform for standardization of care, workforce training, and research that is relevant to the region [51, 52]. Point-of-care diagnostics, handheld ultrasound, artificial intelligence–assisted image interpretation, telemedicine tumor boards, and emerging biomarker platforms including GALAD, and HCC screening assays present promising opportunities for scalable early detection [23, 53, 54]. Community health worker–led screening initiatives, together with the integration of HBV and HCC services into existing HIV and antenatal care frameworks, constitute pragmatic implementation strategies that are particularly well-suited for resource-limited settings.

### Treatment and Outcomes

**Fig. 3:**
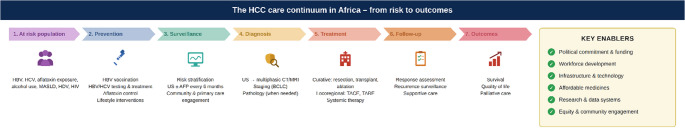
The HCC care continuum, from at-risk population to outcomes. Overview of the seven-stage continuum—at-risk population, prevention, surveillance, diagnosis, treatment, follow-up, outcomes—orienting the framework used throughout this review, expanded with stage-specific gaps and interventions in Figure 2. Core activities are listed beneath each stage (e.g., ultrasound ± AFP for surveillance; BCLC staging with multiphasic CT/MRI for diagnosis). Six cross-cutting enablers—political commitment, workforce development, infrastructure/technology, affordable medicines, research/data systems, equity/community engagement—flank the continuum, reflecting that progress at any stage depends on system-level investment. AFP, α-fetoprotein; BCLC, Barcelona Clinic Liver Cancer; CT, computed tomography; HBV, hepatitis B virus; HCV, hepatitis C virus; MASLD, metabolic dysfunction–associated steatotic liver disease; TACE, transarterial chemoembolization; TARE, transarterial radioembolization; US, ultrasound

The major barriers contributing to poor HCC outcomes across the African care continuum are illustrated in Fig. 3. Outcomes for African patients with HCC remain among the worst globally. Approximately 95% of patients in SSA present with Barcelona Clinic Liver Cancer (BCLC) stage C or D, which precludes them from eligibility for curative treatment options [27, 55]. Diagnosis is usually delayed as patients frequently seek alternative medicines before presenting to formal medical settings. A recent study from Uganda proposed a pragmatic diagnostic approach combining clinical presentation, ultrasound findings, alpha-fetoprotein measurement and pathological assessment to improve diagnostic yield. Despite this approach, median survival following diagnosis was only 46 days [56]. The SURVCAN-3 study, which investigated population-based cancer survival rates in sub-Saharan Africa across 11 countries, reported a three-year net survival of 18.1% for liver cancer [57]. Similarly, a systematic review including 3,989 patients from 39 studies conducted across SSA found that only 6% received any form of curative therapy, whereas 84% received supportive care alone, with almost no survivors beyond one year [55]. The disparities in care across the continent are striking. Within the Africa Liver Cancer Consortium cohort, 76% of patients in Egypt received some form of HCC-specific treatment, compared with only 3% of patients treated in other regions of Africa [4]. This disparity reflects major differences in healthcare resources across the continent. Egypt has established the most active living-donor liver transplantation program in Africa, with broader access to radiofrequency ablation, microwave ablation and transarterial chemoembolization (TACE) through tertiary centers in Cairo and Alexandria. South Africa, the only country in SSA with an established deceased-donor liver transplantation program, also demonstrates the marked impact of healthcare inequity. In a recent comparative cohort, median survival was 68 days for patients managed in the public sector compared with 703 days in the private sector, where access to curative-intent therapies was substantially greater [58]. Across the remainder of SSA, the Africa Guidelines for HCC survey reported liver transplantation availability in only 3% of East and West African centers and none in Central Africa, compared with 28% availability in North and Southern Africa [51]. These disparities are further compounded by late presentation, severe surgical workforce shortages with fewer than two surgeons per 100,000 inhabitants in most SSA countries, limited interventional radiology capacity, and the prohibitive out-of-pocket cost of systemic therapies such as sorafenib, which at approximately USD 5,400 per month exceeds the annual per capita income in many countries across the region [51]. 

Access to curative and life-prolonging therapies in SSA remains highly geographically concentrated. Liver resection is available at a limited number of centers, predominantly located in South Africa, Nigeria, Ethiopia and Sudan. In these regions, selected series have reported five-year survival rates ranging from 38% to 85% among carefully selected patients without cirrhosis [55, 59]. Liver transplantation for HCC in SSA has been reported from a single South African program, in which 31 transplanted patients achieved one- and five-year survival rates of 77% and 61%, respectively [55, 60]. TACE is available in only a limited number of centers. However, it remains largely inaccessible to the majority of patients due to infrastructure limitations and cost barriers [27, 61]. The adoption of systemic therapy remains notably limited. Within the Africa Liver Cancer Consortium cohort, only 12 out of 1,315 patients in SSA (< 1%) received sorafenib, while access to immune checkpoint inhibitors or the atezolizumab–bevacizumab regimen is predominantly unavailable outside tertiary referral centers [4, 27]. Importantly, less than 1% of global HCC clinical trials are conducted in Africa, leading to predictable consequences: local clinicians have limited opportunities to gain experience with novel therapies, and African patients are largely underrepresented in the evidence base that informs international treatment guidelines [27, 51]. This is undoubtedly an area of potential growth as overall, access to medications represent less hurdles than access to loco regional treatments or transplantation.

Encouragingly, the cancer care landscape in Africa is beginning to evolve. Over the past decade, a new generation of regional cancer centers has begun decentralizing oncology services beyond major capital cities. Tanzania exemplifies this shift, expanding from a single national oncology center at Ocean Road Cancer Institute in Dar es Salaam to a broader network that now includes Bugando Medical Centre in Mwanza and Kilimanjaro Christian Medical Centre in Moshi [62, 63]. This expansion has improved access to diagnostic imaging, systemic chemotherapy and selected locoregional therapies for populations that previously traveled long distances for specialist care. Similar decentralization efforts are emerging across Ghana, Kenya, Ethiopia, Rwanda and Uganda. Sustaining and expanding this progress, while deliberately integrating HCC-specific capacity including hepatology expertise, abdominal imaging, interventional radiology and multidisciplinary tumor boards into these emerging centers, remains a major priority of the 2024 IHPBA Legacy Initiative [52]. 

Palliative care, as meaning of prioritizing comfort and quality of life remains significantly underdeveloped in much of Africa. It is estimated that over 80% of patients with cancer die with untreated moderate-to-severe pain [27, 64]. Structured palliative care training programs are currently available in a limited number of countries, including Kenya, Uganda, Botswana, and South Africa [27]. Several barriers impede the widespread implementation of such programs including restrictive opioid regulations, cultural hesitancy regarding opioid use, a shortages of trained specialists, and the low prioritization of palliative services within national cancer control plans [27, 64]. Moreover, patient acceptance and understanding of these cares is low and there is little education in this area [65, 66]. It is imperative to integrate palliative care into routine oncology and hepatology services. Enhancing workforce training in pain management, improving access to essential opioids, and providing psychosocial support for patients and their families can significantly enhance the quality of life, even when a cure is not feasible.

## Conclusion

HCC in Africa is characterized by a high disease burden, younger age at diagnosis, late presentation, and poor survival outcomes. Significant regional heterogeneity remains evident, with HCV being predominant in North Africa and HBV together with aflatoxin exposure driving much of the burden in sub-Saharan Africa. Rising rates of MASLD, harmful alcohol use, and HDV co-infection are additional and under-investigated contributors that are likely to shape the next phase of the disease. The most effective interventions including universal HBV birth-dose vaccination, expansion of HBV and HCV antiviral therapy, aflatoxin mitigation, decentralized community-based surveillance and awareness of unhealthy habits are all technically achievable. Equitable access to curative and systemic therapies, expansion of palliative care, and inclusion of African populations in clinical trials and biomarker discovery are critical areas of improvement for the region.

## Key References

- Butt MA, Aby ES, Debes JD. The global epidemiology of hepatocellular carcinoma. Hepatol Commun. 2026;10:e0932. 10.1097/HC9.0000000000000932.
  - ○ (This contemporary global overview frames the shifting etiologic landscape of HCC and situates Africa’s disproportionate burden within worldwide incidence and mortality trends.)

- Delphin M, Campbell J, Verrier ER, Anderson M, Sukali G, Maponga T, et al. Clinical trials for Hepatitis Delta Virus in the WHO African region: A neglected virus among neglected viruses. J Infect. 2025;91:106514. 10.1016/j.jinf.2025.106514.
  - ○ (This work highlights the near-total absence of HDV clinical research in Africa, underscoring a critical evidence gap for a co-infection that accelerates progression to HCC.)

- Singal AG, Kanwal F, Llovet JM. Global trends in hepatocellular carcinoma epidemiology: implications for screening, prevention and therapy. Nature Reviews Clinical Oncology. Nature Publishing Group; 2023;20:864–84. 10.1038/s41571-023-00825-3.
  - ○ (This review links evolving epidemiologic patterns to actionable screening, prevention, and treatment strategies, providing a framework directly applicable to resource-limited African settings.)

- Chan SL, Sun H-C, Xu Y, Zeng H, El-Serag HB, Lee JM, et al. The Lancet Commission on addressing the global hepatocellular carcinoma burden: comprehensive strategies from prevention to treatment. Lancet. London, England; 2025;406:731–78. 10.1016/S0140-6736(25)01042-6.
  - ○ (This Commission offers a comprehensive prevention-to-treatment roadmap and is foundational for prioritizing interventions across the HCC care continuum in low-resource regions.)

- Ndow G, Vo-Quang E, Shimakawa Y, Ceesay A, Tamba S, Njai HF, et al. Clinical characteristics and outcomes of patients with cirrhosis and hepatocellular carcinoma in The Gambia, west Africa: a prospective cohort study. Lancet Glob Health. 2023;11:e1383–92. 10.1016/S2214-109X(23)00263-2.
  - ○ (This prospective West African study provides high-quality primary data on presentation and dismal outcomes of HCC, quantifying the late-stage diagnosis problem that drives regional mortality.)

- Mann EM, Akambase J, Searle K, Hunt S, Debes JD. Differential Association of Hepatocellular Carcinoma Related to Hepatitis B Between Urban and Rural Areas in Africa Using Satellite Spatial Scaling Data. JCO Glob Oncol. 2025;11:e2400543. 10.1200/GO-24-00543.
  - ○ (This novel spatial analysis reveals urban-rural heterogeneity in HBV-related HCC, informing geographically targeted prevention and surveillance strategies.)

- Sobnach S, Kotze U, Spearman CW, Sonderup MW, Kim I, Venter K, et al. The Natural History and Prognostic Determinants of Untreated Hepatocellular Carcinoma in a Sub-Saharan African Cohort. Journal of Gastrointestinal Cancer. Springer; 2026;57:49. 10.1007/s12029-026-01414-0.
  - ○ (This cohort characterizes the natural history of untreated disease, documenting the survival reality most African patients face in the absence of curative access.)

- Sobnach S, Emmamally M, Venter K, Spearman CW, Kim I, Bernon M, et al. Presentation, treatment and long-term outcomes of hepatocellular carcinoma in patients with and without HIV: a comparative observational cohort study. HPB (Oxford). 2025;27:789–98. 10.1016/j.hpb.2025.02.012.
  - ○ (This comparative study clarifies the influence of HIV co-infection on HCC presentation and outcomes, a clinically important intersection in high-HIV-prevalence settings.)

- Guidelines for the prevention, diagnosis, care and treatment for people with chronic hepatitis B infection [Internet]. Geneva: World Health Organization; 2024 [cited 2026 Apr 24]. http://www.ncbi.nlm.nih.gov/books/NBK614989/. Accessed 24 Apr 2026.
  - ○ (These updated WHO guidelines define the standard of care for chronic HBV and anchor recommendations for prevention interventions central to HCC control.)

- Joko-Fru WY, Bardot A, Bukirwa P, Amidou S, N’da G, Woldetsadik E, et al. Cancer survival in sub-Saharan Africa (SURVCAN-3): a population-based study. Lancet Glob Health. 2024;12:e947–59. 10.1016/S2214-109X(24)00130-X.
  - ○ (This population-based study provides authoritative survival estimates across sub-Saharan cancer registries, benchmarking the poor prognosis of liver cancer against other malignancies.)

- Tang NSY, Gunalan S, Ong CEY, Koh B, Tham EKJ, Lim RY, et al. Meta-Analysis: Utilisation of Hepatocellular Carcinoma Surveillance. Aliment Pharmacol Ther. 2026;63:8–16. 10.1111/apt.70403.
  - ○ (This meta-analysis quantifies the persistent underuse of HCC surveillance, reinforcing why most patients present too late for curative intervention.)

- Heller T, Phiri V, Kumwenda T, Mzumara W, Vinikoor MJ, Rambiki E, et al. Point-of-care ultrasound to inform antiviral treatment initiation in chronic hepatitis B virus infection in low-resource settings - the PUSH protocol. Ultrasound J. 2024;16:18. 10.1186/s13089-024-00369-2.
  - ○ (This protocol demonstrates a pragmatic point-of-care approach to guide HBV management where advanced diagnostics are unavailable, offering a scalable model for decentralized care.)

- Jonas E, Smith M, Kassianides C, Luyirika E, Wendy Spearman C. IHPBA White Paper - The improvement of management pathways and access to care in sub-Saharan Africa for patients with hepatocellular carcinoma. HPB (Oxford). 2025;27:585–90. 10.1016/j.hpb.2025.02.007.
  - ○ (This white paper proposes concrete pathways to strengthen access to HCC care, directly addressing the health-system gaps that perpetuate poor outcomes.)

- Abou-Alfa GK, Afihene M, Capanu M, Li Y, Chou JF, Asombang A, et al. Africa Guidelines for Hepatocellular Carcinoma Buildup Process. JCO Glob Oncol. 2023;9:e2300159. 10.1200/GO.23.00159.
  - ○ (This effort details the development of Africa-specific HCC guidelines, marking a foundational step toward contextually appropriate management standards for the continent.)

## Data Availability

No datasets were generated or analysed during the current study.

## Abbreviations

Abbreviation: Definition
AFP: Alpha-fetoprotein
APRI: Aspartate Aminotransferase-to-Platelet Ratio Index
ASIR: Age-standardized incidence rate
BCLC: Barcelona Clinic Liver Cancer
CT: Computed tomography
FIB-4: Fibrosis-4 Index
GALAD: Gender, Age, AFP-L3, AFP, and Des-gamma-carboxy Prothrombin Score
GHASSA: Gastroenterology and Hepatology Association of Sub-Saharan Africa
HBsAg: Hepatitis B surface antigen
HBV: Hepatitis B virus
HCC: Hepatocellular carcinoma
HCV: Hepatitis C virus
HDV: Hepatitis D virus
HIV: Human immunodeficiency virus
HCC: Hepatocellular carcinoma
IHPBA: International Hepato-Pancreato-Biliary Association
LI-RADS: Liver Imaging Reporting and Data System
MASLD: Metabolic Dysfunction-Associated Steatotic Liver Disease
MRI: Magnetic resonance imaging
POC: Point-of-care
PUSH: Point-of-care Ultrasound for Staging Hepatitis B (Protocol)
RFA: Radiofrequency ablation
SSA: Sub-Saharan Africa
TACE: Transarterial chemoembolization
TARE: Transarterial radioembolization
US: Ultrasound
WHO: World Health Organization
